# HPLC-HRMS and interpretable machine learning decipher serum lipidomic signatures in NSCLC

**DOI:** 10.7717/peerj.21504

**Published:** 2026-07-08

**Authors:** Chengxi Tang, Jiahua Lyu, Jianming Huang, Xue Zhang, Chunxiao Mou, Yuxin Liu, Shuang Ni, Yudi Liu, Linjie Li, Ling Xiao, Shichuan Zhang, Xiumei Zheng

**Affiliations:** 1 Department of Radiation, Sichuan Clinical Research Center for Cancer, Sichuan Cancer Hospital & Institute, Sichuan Cancer Center, School of Medicine, University of Electronic Science and Technology of China, Chengdu, China; 2 Department of Radiation, Precision Radiation in Oncology Key Laboratory of Sichuan Province, Sichuan Clinical Research Center for Cancer, Sichuan Cancer Hospital, Chengdu, Sichuan, China; 3 Sichuan Clinical Research Center for Cancer, Sichuan Cancer Hospital & Institute, Sichuan Cancer Center, Affiliated Cancer Hospital of University of Electronic Science and Technology of China, University of Electronic Science and Technology of China, Chengdu, Sichuan, China; 4 Department of Oncology, The Affiliated Hospital of Southwest Medical University, Luzhou, Sichuan, China; 5 Chengdu Medical College, Chengdu, Sichuan, China

**Keywords:** Machine learning, Non-small cell lung cancer, Serum lipidomics, Liquid chromatography-mass spectrometry

## Abstract

**Background:**

Non-small cell lung cancer (NSCLC) remains the leading cause of cancer mortality, largely due to the lack of reliable non-invasive tools for detection and risk stratification. Lipid metabolic reprogramming is a hallmark of cancer and may serve as a promising source of diagnostic biomarkers.

**Methods:**

Serum from 40 NSCLC patients and 30 controls was profiled by high-performance liquid chromatography—high-resolution mass spectrometry (HPLC-HRMS), quantifying 331 annotated lipids. Differential and pathway analyses were performed. Least absolute shrinkage and selection operator (LASSO), support vector machine (SVM), Extreme Gradient Boosting (XGBoost), and Light Gradient-Boosting Machine (LightGBM) models were evaluated using stratified 10-fold cross-validation; feature prioritization used recursive feature elimination and Shapley additive explanations (SHAP). A combined clinical–lipid model incorporating selected lipids and clinical covariates was assessed with discrimination, calibration, and decision-curve analysis.

**Results:**

NSCLC exhibited broad decreases in glycerophospholipids, sphingolipids, and triacylglycerols, consistent with membrane-lipid remodeling. LightGBM showed the best discrimination in internal validation. Key discriminant lipids included lysophosphatidylcholine (LPC(O-18:1)), decanoylcarnitine, and sulfatide (SL) (SL 38:5). The integrated lipid–clinical model achieved good discrimination (area under the receiver operating characteristic curve (AUC) = 0.946) and acceptable calibration. A nomogram was constructed for individualized risk estimation.

**Conclusions:**

This study nominates candidate serum lipid markers and an interpretable modeling workflow for NSCLC classification in an exploratory case–control cohort. External validation and targeted quantification in larger, multicenter and screening-relevant populations are required before clinical implementation.

## Introduction

Lung cancer remains the leading cause of cancer-related mortality worldwide, accounting for approximately 1.8 million deaths in 2020 according to GLOBOCAN (Sung et al., 2021). Its high lethality is largely attributable to the difficulty of early detection. Non-small cell lung cancer (NSCLC) accounts for approximately 80–85% of all lung cancer cases, with lung adenocarcinoma and squamous cell carcinoma being the predominant histological subtypes (Goodwin et al., 2017). Although therapeutic options for NSCLC have expanded (including surgery, radiotherapy, systemic chemotherapy, targeted therapy, and immunotherapy), their survival benefit remains highly contingent on stage at diagnosis. As early disease is often asymptomatic or presents with nonspecific symptoms, approximately 60–70% of patients are still diagnosed at locally advanced or metastatic stages (Ganti et al., 2021), resulting in a dismal overall 5-year survival rate of less than 20% (Zappa & Mousa, 2016). Accordingly, shifting diagnosis toward earlier stages expands access to curative-intent interventions and is associated with substantially improved survival, underscoring the clinical value of accurate early detection. Although low-dose computed tomography (LDCT) screening has been shown to reduce cancer mortality, its utility is limited by high false-positive rates—approximately 20% on average and up to 27% in early screening rounds of the National Lung Screening Trial (NLST), radiation exposure, and cost-effectiveness concerns (Wade et al., 2018). Conventional serum tumor markers such as carcinoembryonic antigen (CEA) and neuron-specific enolase (NSE) exhibit suboptimal diagnostic performance in early-stage disease (Arrieta et al., 2021). Hence, the development of non-invasive and sensitive molecular biomarkers is crucial to improve early detection and patient outcomes.

Metabolic reprogramming, including alterations in lipid metabolism, has been recognized as a hallmark of cancer (Pavlova & Thompson, 2016; Hanahan & Weinberg, 2011). Lung cancer cells actively remodel key lipid classes—including glycerophospholipids, sphingolipids, and triglycerides—to sustain rapid proliferation and survival (Cheng et al., 2018; Beloribi-Djefaflia, Vasseur & Guillaumond, 2016). These alterations suggest that circulating lipid profiles may provide molecular signatures for early diagnosis. Lipidomics, an emerging branch of metabolomics, enables comprehensive characterization of lipid species through high-resolution mass spectrometry and has shown unique advantages in biomarker discovery and mechanistic studies (Quehenberger & Dennis, 2011). Prior investigations have revealed marked disturbances in serum lipid profiles of lung cancer patients, which correlate with tumor stage, histological subtype, and aggressiveness, thereby underscoring their potential utility as diagnostic biomarkers (Wang et al., 2022; Lv et al., 2018). However, most of the lipidomics studies remain limited to preliminary screening, lack systematic model construction and interpretability, hindering clinical translation (Zhang et al., 2020).

High-performance liquid chromatography–high-resolution mass spectrometry (HPLC-HRMS) provides a high-throughput, broad-coverage, and accurate platform for lipid quantification, allowing simultaneous profiling of thousands of lipid species in complex biological samples (Munjoma et al., 2022). Nevertheless, MS-based lipidomic datasets are typically characterized by high dimensionality, collinearity, and feature redundancy, which challenge conventional statistical approaches in feature selection and model stability (Morabito et al., 2025). Machine learning (ML) techniques have thus emerged as powerful tools for lipidomic modeling. Classical supervised algorithms such as least absolute shrinkage and selection operator (LASSO) regression, support vector machine (SVM), extreme gradient boosting (XGBoost), and light gradient-boosting machine (LightGBM) have demonstrated promising performance in blood-based biomarker studies across multiple cancer types (Liu et al., 2025; Zhou et al., 2022). Moreover, model-interpretability tools such as shapley additive explanations (SHAP) provide quantitative, instance-level attributions of feature contributions, enabling both local and global understanding of complex models and thereby enhancing transparency and clinical credibility (Lundberg et al., 2020).

In this exploratory case–control study with a modest sample size, we hypothesized that NSCLC is associated with a discernible serum lipidomic perturbation reflecting membrane lipid remodeling, and that an interpretable machine-learning framework can summarize these high-dimensional profiles to discriminate NSCLC cases from controls under internal resampling. Serum samples from lung cancer patients and controls were prospectively analyzed by HPLC-HRMS to comprehensively characterize lipidomic alterations. Key lipid candidates were prioritized using LightGBM, LASSO, and related algorithms, and subsequently combined with clinical variables into multivariable models. Model performance was evaluated using SHAP-based interpretability and decision curve analysis (DCA). We emphasize that the current study is not a screening validation study; independent evaluation in larger, multi-center cohorts is required to determine generalizability and clinical applicability for early detection.

## Materials and Methods

### Study population and serum acquisition

A total of 70 participants were included, comprising 40 patients with histologically confirmed NSCLC and 30 age- and sex-comparable control participants. All subjects were recruited at Sichuan Cancer Hospital. Demographic and clinical variables (*e.g.*, age, body mass index [BMI], smoking history) were recorded. For both NSCLC patients and controls, venous blood was collected after overnight fasting using the same standard venipuncture procedure and collection tubes. NSCLC samples were obtained prior to any anti-tumor treatment, whereas control samples were collected during routine health check-ups; no additional procedures were performed for NSCLC patients beyond routine blood collection. Samples were processed using an identical standardized protocol: blood was processed within 1 h of collection, followed by centrifugation at 3,000 rpm for 15 min at 18 °C, immediate serum aliquoting and storage at −80 °C until analysis. Written informed consent was obtained from all participants, and the study was approved by the Ethics Committee of Sichuan Cancer Hospital (SCCSMC-01-2024-254).

### Isolation of serum lipids

For lipid extraction, 20 µL of serum was mixed with 10 µL of internal standard solution (PC 19:0/19:0, 10 µg/mL in methanol) and vortexed thoroughly. A total of 640 µL of methanol/MTBE (140:500, v/v) was then added, and the mixture was agitated for 10 min to facilitate lipid release. To induce phase separation, 150 µL of ultrapure water was introduced, followed by vortexing and centrifugation at 14,000 rpm for 10 min. The resulting upper organic fraction (200 µL) was collected, evaporated to dryness using a vacuum concentrator, and stored at −80 °C until analysis. Prior to LC–MS measurement, dried extracts were re-dissolved in 200 µL of acetonitrile/isopropanol/water (65:30:5, v/v/v), vortexed, and centrifuged at 14,000 g for 15 min at 18 °C. A 40 µL aliquot of the clear supernatant was transferred to autosampler vials. In addition, a pooled quality control (QC) sample was generated by combining equal volumes from all study specimens. All preparations were handled in a single analytical batch to minimize variation.

### Comprehensive lipid profiling using LC-Orbitrap MS

Untargeted lipidomic profiling was conducted on a Dionex Ultimate 3000 UHPLC system coupled with a Q-Exactive Plus Orbitrap mass spectrometer (Thermo Fisher Scientific). Chromatographic separation was achieved on a Hypersil GOLD C18 column (100 × 2.1 mm, 1.9 µm) maintained at 45 °C. The mobile phases consisted of solvent A (acetonitrile/water, 60:40, v/v, with 0.1% formic acid and 10 mM ammonium formate) and solvent B (isopropanol/acetonitrile, 90:10, v/v, with 0.1% formic acid and 10 mM ammonium formate). The flow rate was set at 0.3 mL/min, and the elution program was as follows: 0–4 min, 15% B; 4–6 min, 15–45% B; 6–22 min, 45–85% B; 22–23 min, 85–99% B; 23–26 min, 99% B; 26–26.1 min, 99–15% B; 26.1–30 min, 15% B. Mass spectrometry was carried out in both positive and negative heated electrospray ionization (HESI) modes, using spray voltages of +3.5 kV and −3.0 kV, respectively. The ion source parameters were as follows: capillary temperature, 320 °C; sheath gas, 45 (arbitrary units); auxiliary gas, 10 (arbitrary units); S-lens RF level, 50. Full-scan MS data were recorded with a resolving power of 70,000 over an m/z range of 250–1,500 (positive mode) and 200–1,500 (negative mode), with an AGC target of 1 × 10^6^ and a maximum injection time of 100 ms. Data-dependent MS^2^ acquisition was performed on the top 10 precursor ions, with a resolution of 17,500, AGC target of 1 × 10^5^, maximum injection time of 50 ms, isolation window of 1.0 m/z, stepped collision energies of 25% and 30%, and dynamic exclusion of 10 s. The injection volume was 5 µL for all analyses. MS/MS spectra for identification were acquired using DDA primarily on repeated pooled-QC injections.

### Lipidomic identification and data processing

Raw LC–MS data were annotated using Compound Discoverer 3.3 (Thermo Fisher Scientific) based on a combination of accurate mass, retention time, and MS/MS spectral information. High-resolution precursor ions were matched to theoretical masses with a tolerance of ±5.00 ppm, and isotopic patterns were evaluated to confirm elemental composition. Tandem mass spectra (MS2) were acquired in data-dependent acquisition (DDA) mode, where preferred precursor ions were selected for fragmentation to ensure reliable annotation of target lipids. Characteristic fragment ions, neutral losses, and headgroup-specific signals were used to discriminate lipid subclasses and to minimize misannotation from isobaric species. Spectral matching was performed against multiple reference databases, including mzCloud, mzVault, Metabolika, and ChemSpider. Candidate exact-mass matches were generated using a MassList derived from the LIPID MAPS Structure Database (LMSD). For data quality control, features were retained only when at least six “usable” QC samples were available for quantification (*n* ≥ 6) and the relative standard deviation (RSD) across QC replicates was ≤ 20%; features failing either criterion were removed prior to downstream analyses. Peak intensities were normalized to the internal standard PC 19:0/19:0, log_2_(x+1) transformation was applied to stabilize variance, and values were subsequently *z*-score standardized to enhance comparability across samples. Non-lipid features and features with missing values across samples were removed prior to downstream statistical analysis and modeling.

### Sample randomization and quality control (QC) strategy

Serum samples were assigned anonymized codes and the LC-MS injection order of study samples was randomized using a random-number sequence prior to data acquisition. A pooled QC sample was prepared by combining equal aliquots of serum from all study participants and was injected repeatedly under the same LC-MS conditions as study samples to monitor analytical stability and support QC-based signal-drift correction. The analytical sequence started with one solvent blank injection, followed by three pooled QC injections for system conditioning. Subsequently, study samples were acquired in blocks of six injections, with one pooled QC injection inserted after every six study samples (QC:sample ratio = 1:6) throughout the entire run until completion.

### Machine learning modeling and evaluation

All statistical analyses and machine learning modeling were performed in R (version 4.5.0). Model development used a nested resampling strategy with an outer 10-fold stratified cross-validation (CV) to generate out-of-fold (OOF) predictions. Within each outer training fold, features were standardized using the training-fold mean and standard deviation and then applied to the corresponding test fold to prevent information leakage. LASSO logistic regression selected the optimal *λ via* inner 10-fold CV (cv.glmnet). For XGBoost and LightGBM, the best boosting iteration was determined within each outer training fold using 10-fold stratified CV with log-loss and early stopping, followed by refitting on the full outer training set and predicting the held-out fold. SVM models were tuned within the outer training folds using inner CV optimizing ROC AUC; for the RBF kernel, C and kernel width (sigma, corresponding to *γ*) were tuned by grid search. Performance was assessed using OOF probabilities, including ROC/AUC with DeLong 95% CI, Youden’s index, accuracy, sensitivity, specificity, PPV, NPV, F1 score, and Brier score. Stratified bootstrap resampling (*B* = 1,000) on OOF predictions was used to estimate the AUC distribution. Complete scripts are provided in the Supplementary Information.

### Feature selection and model interpretation

All analyses were conducted in R using readxl, matrixStats, limma, caret, lightgbm, and pROC. All preprocessing, standardization, and feature selection were performed within each training fold only, and the learned transformations were then applied to the corresponding held-out fold. Within each outer split, candidate discriminatory lipids were identified using limma with empirical Bayes moderation, comparing cases *versus* controls in the training set; lipids were retained if they met Benjamini–Hochberg FDR < 0.05 and an effect-size criterion defined as the absolute standardized mean difference between groups in z-space. The resulting fold-specific lipid subset was then used to train a LightGBM binary classifier under stratified 10-fold cross-validation, generating out-of-fold predicted probabilities for every participant. LightGBM models were fit with objective = “binary” and metric = “auc”, using the following hyperparameters: learning_rate = 0.05, num_leaves = 31, min_data_in_leaf = 10, feature_fraction = 0.90, bagging_fraction = 0.80, bagging_freq = 1, max_depth = −1, verbosity = −1, and feature_pre_filter = FALSE; Training was run for up to 3,000 boosting iterations with early stopping (rounds = 150) based on validation AUC within each fold, and the fold-specific best iteration number was recorded; overall discrimination was summarized by an ROC curve and AUC computed from the aggregated out-of-fold predictions, with 95% confidence intervals estimated by DeLong’s method. A final LightGBM model was refit on the full dataset using a cross-validated estimate of the optimal boosting iterations, and SHAP values were computed using TreeSHAP *via* LightGBM’s contribution predictions. Global feature importance was summarized as the mean absolute SHAP value per lipid, and sample-level effects were visualized using SHAP beeswarm plots to quantify both the magnitude and direction of each lipid’s contribution to the model output.

### Predictive modeling and validation

Three feature sets were assessed: Model A (lipids), Model C (clinical only) and Model B (Model A + age, BMI, binary drink history); outcome coded lung cancer = 1, control = 0. Stratified 10-fold cross-validation with fold-wise *z*-scoring was applied, and LightGBM with internal cross-validation and early stopping generated out-of-fold probabilities. Performance was summarized by AUC (95% CI) with paired DeLong comparison; calibration used adaptive binning with weighted logistic smoothing. Clinical utility was evaluated by decision-curve analysis with Treat-All/Treat-None references, and an interpretable surrogate (ridge-penalized logistic regression with restricted cubic splines) supported nomogram construction. All analyses were conducted in R (version 4.5.0).

## Results

### Serum lipid class composition and subclass-specific alterations in NSCLC

**Figure 1:**
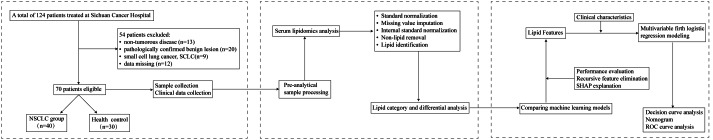
Study cohort selection and experimental workflow. Flowchart illustrating patient inclusion and exclusion criteria, serum collection and lipidomics analysis, followed by bioinformatics and machine learning–based modeling and evaluation.

To identify NSCLC-specific lipidomic signatures, we prospectively collected serum samples from 40 pathologically confirmed NSCLC patients and 30 controls (total *n* = 70) between September and December 2024. Lipidomic profiling was performed using high-performance liquid chromatography–high-resolution mass spectrometry (HPLC-HRMS), with rigorous preprocessing and normalization of raw data. The cohort selection strategy and experimental workflow are illustrated in Fig. 1, and demographic and clinical characteristics of the study participants are summarized in Table 1.

**Figure 2:**
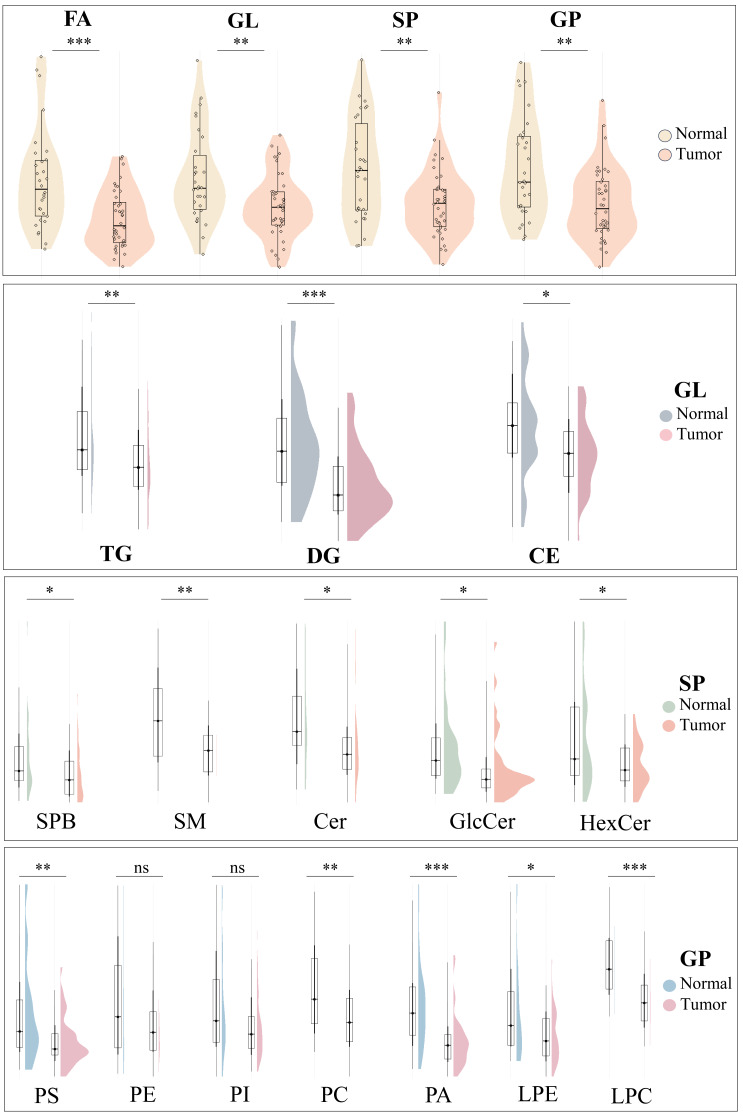
Comparison of serum lipid classes between lung cancer and controls. Violin plots show the distributions of lipid classes (FA, GL, SP, GP) and representative subclasses (TG, DG, CE; SPB, SM, Cer, GlcCer, HexCer; PS, PE, PI, PC, PA, LPE, LPC) in NSCLC (Tumor) *versus* Normal controls. Statistical significance was assessed by two-sided Student’s *t*-test: *P* < 0.05, *P* < 0.01, ^∗^*P* < 0.001; ns, not significant.

A total of 331 distinct lipid species were detected through serum lipidomic analysis of NSCLC patients and controls. Based on Lipid Maps classification and structural features, these were categorized into four major classes: fatty acids (FA, *n* = 9, 2.72%), glycerolipids (GL, *n* = 95, 28.70%), glycerophospholipids (GP, *n* = 171, 51.66%), and sphingolipids (SP, *n* = 56, 16.92%). Subclass annotation included glycerolipids (GL; triacylglycerols (TG), diacylglycerols (DG), cholesteryl esters (CE)), glycerophospholipids (GP; phosphatidylserine (PS), phosphatidylethanolamine (PE), phosphatidylinositol (PI), phosphatidylcholine (PC), phosphatidic acid (PA), lysophosphatidylethanolamine (LPE), and lysophosphatidylcholine (LPC)), and sphingolipids (SP; sphingoid bases (SPB), sphingomyelins (SM), ceramides (Cer), glucosylceramides (GlcCer), and hexosylceramides (HexCer)), as illustrated in Fig. 2. Overall, significant compositional differences were observed between the NSCLC and control groups. All lipid classes showed marked reductions in the NSCLC group, indicating widespread lipid metabolic reprogramming. Within glycerophospholipids, PS, PC, PA, LPE and LPC were decreased, whereas PE and PI showed no significant differences. These findings indicate a broad reduction of serum lipids in the tumor group, with subclass-specific magnitudes of decrease (most pronounced for triacylglycerols, sphingolipids, and choline/acidic glycerophospholipids), consistent with lipid metabolic reprogramming and potential shifts in lipid-class composition.

### Differential serum lipids and their clinical associations

**Figure 3:**
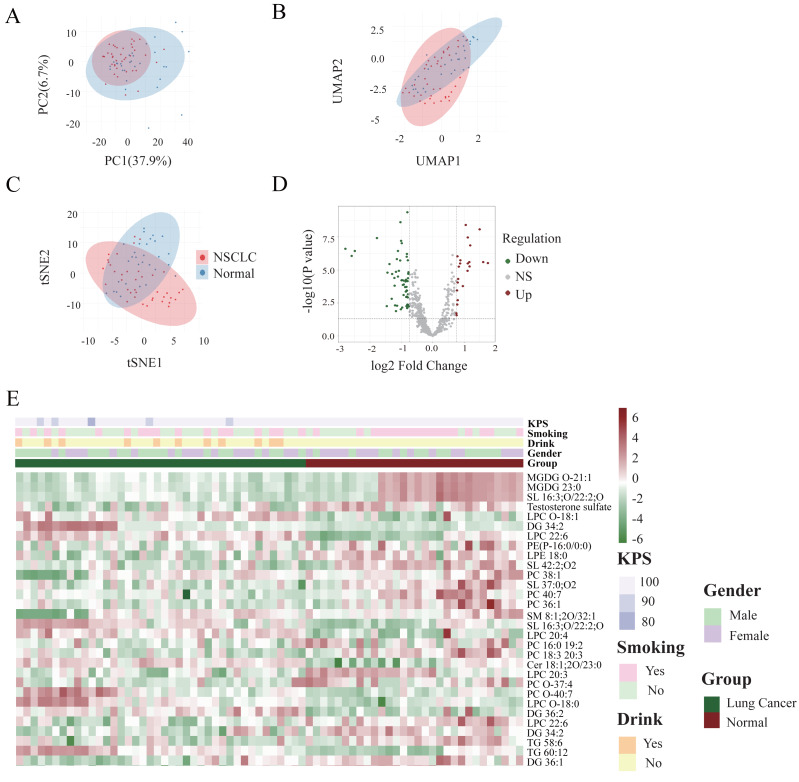
Multidimensional analyses of serum lipidomic differences between lung cancer patients and controls. (A–C) Unsupervised dimensionality reduction analyses using (A) Principal Component Analysis (PCA). (B) Uniform Manifold Approximation and Projection (UMAP), and (C) t-distributed Stochastic Neighbor Embedding (t-SNE). (D) Volcano plot revealed differentially expressed lipids between LC and NC group. (E) Heatmap of differential lipids with hierarchical clustering across samples, annotated with clinical variables (KPS score, smoking, drinking, and gender).

To investigate overall lipidomic divergence, we applied three unsupervised dimensionality reduction methods—PCA, UMAP, and t-SNE—to compare NSCLC and control samples, as shown in Figs. 3A–3C. PCA revealed partial separation between groups along the first principal component (PC1), which explained 37.9% of total variance, with NSCLC samples clustering more tightly. UMAP preserved both local and global data structures and further enhanced group separation, while t-SNE likewise demonstrated clear spatial segregation between NSCLC and control samples. As an auxiliary supervised analysis, we additionally performed OPLS-DA, and the corresponding score plot and permutation analysis are provided in the Supplementary Material. Using *t*-tests with FDR correction (adjusted *P* < 0.05, —log2FC— > 0.75), we identified 75 differentially abundant lipids, of which 53 were decreased and 22 were increased in NSCLC relative to controls (Fig. 3D). The heatmap provided a visual overview of the expression patterns of these lipids and showed group-level differences between NSCLC and HC (Fig. 3E). Overall, these results support the presence of broad lipidomic differences between NSCLC patients and healthy controls.

### Pathway enrichment analysis identifies dysregulation of membrane lipid metabolism

**Figure 4:**
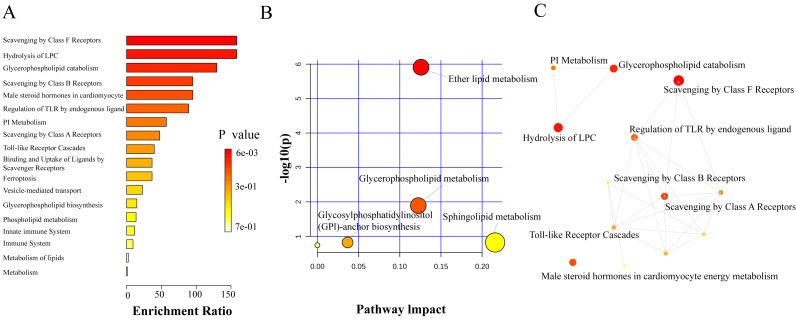
Functional enrichment and pathway analysis of differential serum lipids. (A) Bar plot of metabolite set enrichment analysis showing significantly enriched lipid-related pathways, ranked by adjusted *p*-values. (B) Bubble plot of pathway analysis based on the Kyoto Encyclopedia of Genes and Genomes (KEGG) database, with bubble size representing pathway impact and color indicating significance level. (C) Network topology visualization of enriched pathways highlighting the interconnections among lipid metabolism pathways.

To explore the biological functions of differential lipids, enrichment and pathway analysis were performed using MetaboAnalyst 6.0, as shown in Fig. 4. Altered lipids were primarily enriched in hydrolysis of LPC, glycerophospholipid metabolism, phosphatidylinositol (PI) metabolism, and glycerophospholipid biosynthesis pathways. Pathway impact analysis further highlighted glycerophospholipid metabolism, ether lipid metabolism, and sphingolipid metabolism as the most perturbed pathways. In addition, network topology analysis demonstrated close interconnections among these metabolic pathways. Overall, these pathway-level analyses pointed to coordinated alterations in membrane-associated lipid metabolism in NSCLC.

**Table 1:** Baseline characteristics of patients with lung cancer (LC) and controls (NC).

	*LC(n* = 40*)*^a^	*NC(n* = 30*)*^a^	*p*-value
Age, mean ± sd	59.85 ± 9.83	63.80 ± 8.78	0.0815^b^
BMI, *n*, (%)			0.3348^d^
<25.0	16 (40.0%)	16(53.3%)	
≥ 25.0	24 (60.0%)	14(46.7%)	
Sex(male/female)	23/17	14/16	0.5114^c^
Smoking, *n*, (%)	17(42.5%)	18 (60.0%)	0.227^c^
Current	10	9	
Non-current	7	9	
Alcohol drinking, *n*, (%)	11(27.5%)	0 (0%)	–
Height (m)	1.60 ± 0.07	1.67 ± 0.08	–
Weight (kg)	58.16 ± 9.05	69.70 ± 4.61	–
KPS, *n*, (%)			
100	35 (87.5%)	30 (100%)	
90	4 (10.0%)	–	–
80	1 (2.5%)	–	–
CEA (ng/mL)	2.28 (1.38–4.31)	–	–
SCC (ng/mL)	0.66 (0.52–1.31)	–	–
CYFRA21 -1 (ng/mL)	3.36 (2.13–6.54)	–	
TC (mmol/L)	4.42 (3.89–5.11)	–	–
TG (mmol/L)	1.14 (0.92–1.72)	–	–
ALB (g/L)	38.60 (35.80–41.45)	–	–
PLT (×10^9^ /L)	205.00 (155.25–254.25)	–	–
NEU (×10^9^ /L)	3.58 (2.28–5.33)	–	–
LYM (ng/mL)	1.56 (1.34–1.83)	–	–
HB (g/L)	130.0 (121.75–139.25)	–	–
Pathology		–	–
LUSC	13 (32.5%)	–	–
LUAD	27 (67.5%)	–	–
Clinical T stage		–	–
1	11 (27.5%)	–	–
2	8 (20.0%)	–	–
3	11 (27.5%)	–	–
4	10 (25.0%)	–	–
Clinical N stage		–	–
0	10 (25.0%)	–	–
1	10 (25.0%)	–	–
2	12 (30.0%)	–	–
3	8 (20.0%)	–	–
Metastasis		–	–
M0	32 (80.0%)	–	–
M1	8 (20.0%)	–	–
Stage, *n*, (%)		–	–
I	13 (32.5%)	–	–
II	6 (15.0%)	–	–
III	10 (25.0%)	–	–
IV	11 (27.5%)	–	–
Tumor Size		–	–
≤ 5 cm	33 (82.5%)	–	–
>5 cm	7 (17.5%)	–	–

**Notes.**

a Mean (SD) n (%); Median (IQR).

b Two sample *t*-test.

c Pearson’s Chi-square test.

d Fisher’s exact test.

### Diagnostic performance of machine learning models and SHAP-based interpretation

**Figure 5:**
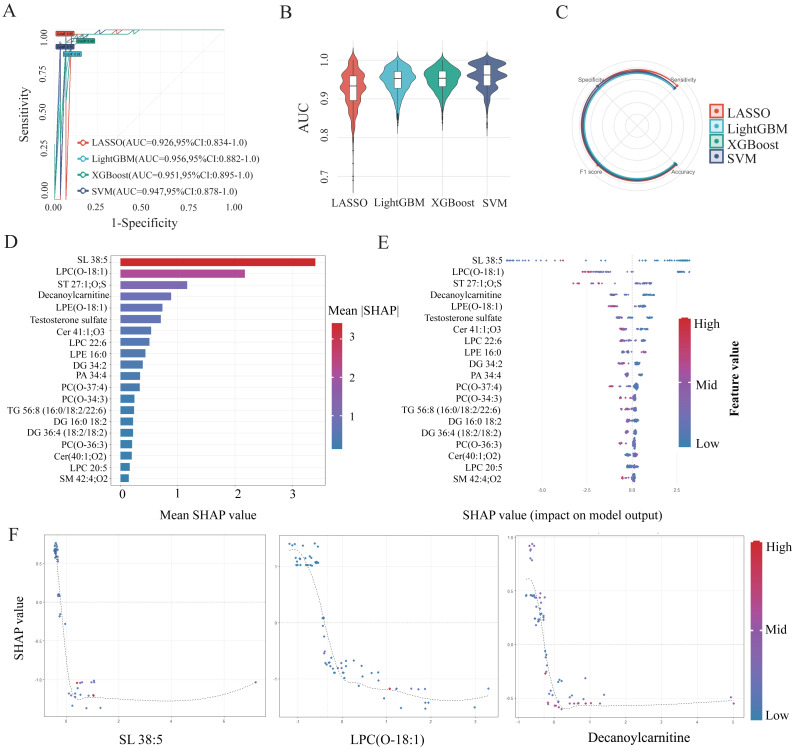
Machine learning model performance and SHAP-based feature interpretation for distinguishing NSCLC from NC. (A) Receiver operating characteristic (ROC) curves of four models (LASSO, LightGBM, SVM, and XGBoost) with area under the curve (AUC) and best cut-off points indicated. (B) Violin plots showing the bootstrap distribution of AUC values across 1,000 resamplings for each model. (C) Radar chart comparing overall diagnostic performance (accuracy, sensitivity, specificity, and F1 score) of the four models. (D) Bar plot of the top lipid features ranked by mean absolute SHAP values. (E) SHAP beeswarm plot illustrating the distribution of SHAP values for the top lipid features across individual samples. (F) SHAP dependence plots for the selected lipid features (LPC(O-18:1), Decanoylcarnitine and SL 38:5).

To evaluate the diagnostic value of differential lipids, four machine learning classifiers (LASSO, LightGBM, XGBoost, and SVM) were implemented within an outer 10-fold stratified cross-validation framework. As shown in Fig. 5A, receiver operating characteristic (ROC) analysis indicated strong discriminative performance across most models. LightGBM achieved the highest AUC (0.956; 95% CI [0.882–1.000]), followed by XGBoost (0.951; 95% CI [0.895–1.000]) and SVM (0.947; 95% CI [0.878–1.000]), while LASSO showed a slightly lower AUC (0.926; 95% CI [0.834–1.000]). Bootstrap resampling (*B* = 1,000) further supported the stability of these estimates (Fig. 5B), with LightGBM and XGBoost exhibiting relatively tighter AUC distributions than LASSO and SVM. Comparative diagnostic metrics (Fig. 5C and Table 2) further showed that all models achieved strong overall classification performance, with accuracy ranging from 0.914 to 0.957 and consistently high specificity. Sensitivity varied across algorithms, with LASSO and XGBoost reaching 0.975, SVM 0.950, and LightGBM 0.900. Calibration was generally good, with low Brier scores (0.069–0.075). Taken together, these results suggest that no single model dominated every metric: LightGBM provided the highest discriminative ability (AUC), whereas LASSO achieved the highest accuracy and the lowest Brier score. We therefore prioritised LightGBM as the primary model for subsequent analyses based on its top AUC and stable performance in bootstrap resampling, while acknowledging the trade-offs observed in threshold-dependent metrics.

The top discriminative lipid features are presented in Fig. 5D. Among them, the sulfonolipid (SL) 38:5 and LPC(O-18:1) showed the greatest contributions, followed by the sterol lipid (ST) 27:1;O;S, Decanoylcarnitine, and several lipid species from LPE and PC, Cer, and DG and TG subclasses. The beeswarm plot further indicates that most key features yield positive SHAP values at low abundance—thereby increasing the predicted probability of lung cancer (positive class)—which aligns with the class/subclass-level downward trends described above (Fig. 5E). To improve interpretability, recursive feature elimination (RFE) was applied within the LightGBM framework, followed by SHAP analysis. SHAP dependence plots for the selected features further corroborated these trends, reinforcing their role as key lipid biomarkers for disease discrimination (Fig. 5F). Representative lipid biomarkers identified through LightGBM modeling are summarized in Table 3.

### Performance of the integrated lipid–clinical model in internal cross-validation

**Figure 6:**
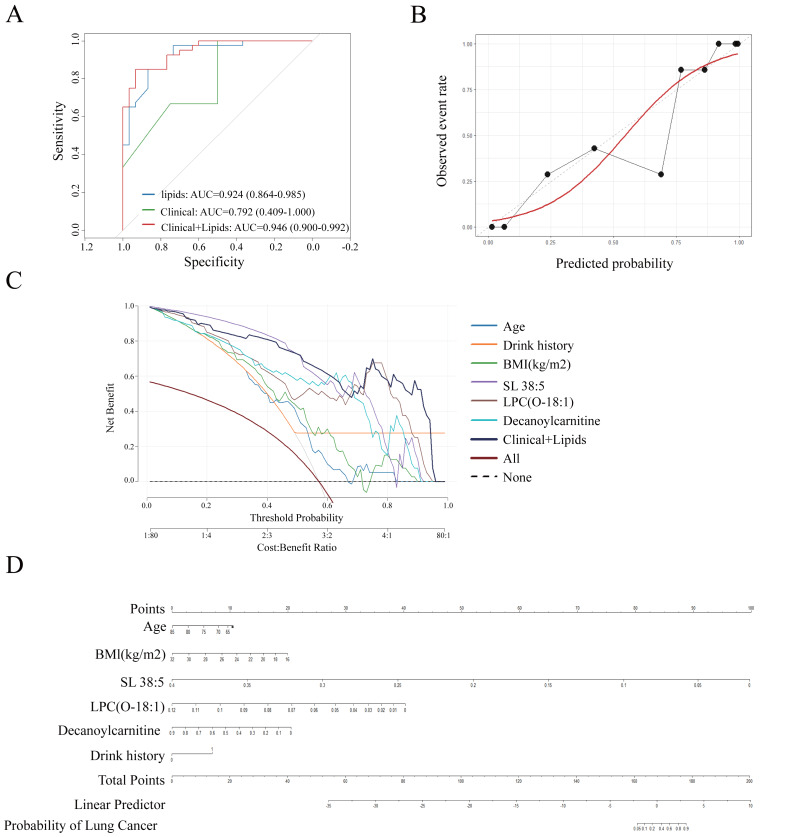
Performance evaluation of the multivariable firth logistic regression model integrating lipidomic and clinical features. (A) ROC curves comparing the lipid-only, Clinical and Clinical+Lipids models. (B) Decile-binned calibration plot with LOESS smoothing. (C) Decision curve analysis (DCA). (D) Nomogram constructed from the multivariable Firth logistic regression model, integrating age, drinking history, BMI, and three key lipid features.

The top 20 lipid features ranked by SHAP importance were incorporated with clinical variables (sex, age, smoking history, drink history, and BMI) into Firth logistic regression analysis. Age, drink history, BMI and three lipids (SL 38:5, LPC(O-18:1), Decanoylcarnitine) were significantly associated with lung cancer risk, and multivariable analysis confirmed their independent predictive value. As shown in Fig. 6A, the integrated clinical–lipid model achieved the highest discriminative performance (AUC = 0.946; 95% CI [0.900–0.992]), outperforming the lipid-only model (AUC = 0.924; 95% CI [0.864–0.985]) and the clinical-only model (AUC = 0.792; 95% CI [0.409–1.000]) for distinguishing NSCLC cases from controls. Out-of-fold calibration using adaptive binning with weighted logistic smoothing showed good agreement between predicted and observed risks (Fig. 6B). The model yielded a Brier score of 0.103 and a scaled Brier score of 0.580, supporting the reliability of the probability estimates. Decision-curve analysis showed that the integrated model yielded greater net benefit than the reference strategies (treat-all, treat-none) and any single-variable model across threshold probabilities of approximately 0.10–0.80 (Fig. 6C). Finally, a nomogram incorporating age, drink history, BMI, and the three lipids was developed, providing a practical tool for individualized risk prediction and potential clinical application (Fig. 6D).

### Subgroup-specific lipidomic profiles reflect clinical heterogeneity of NSCLC

**Figure 7:**
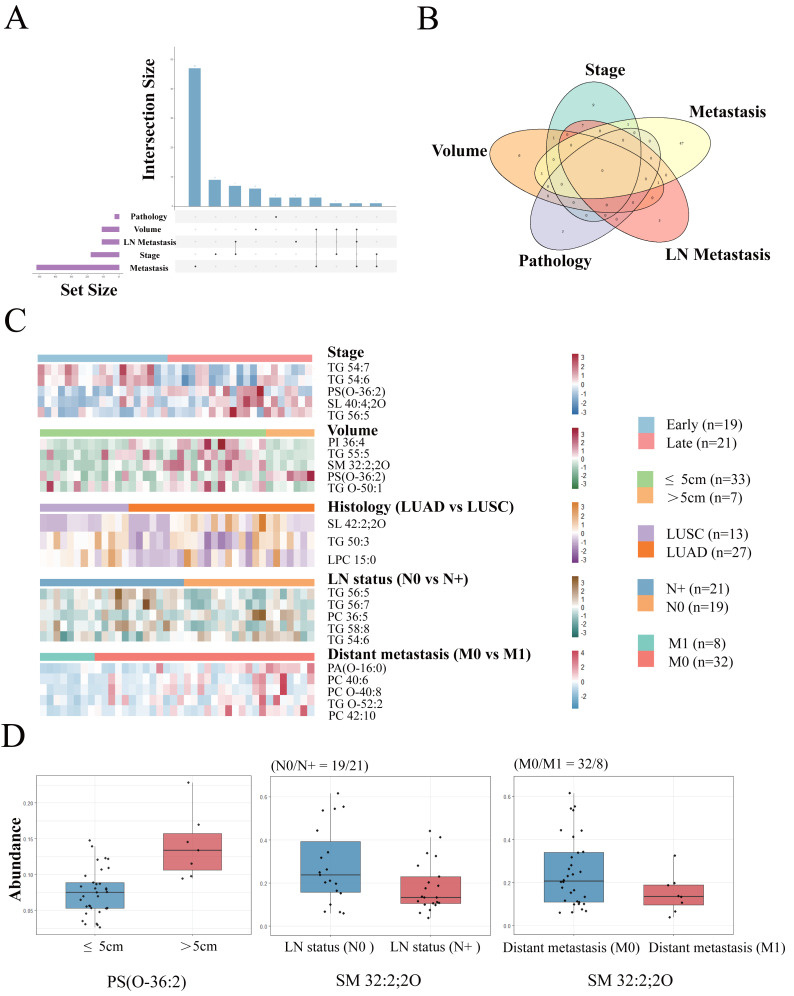
Differential lipid signatures across lung cancer subgroups. (A) Upset plot of unique and shared differential lipids among clinical subgroups (stage, tumor size, histology (LUAD *vs* LUSC), regional lymph-node involvement, and distant metastasis). (B) Venn diagram showing overlapping lipids across stage, tumor size, and metastasis, suggesting potential core biomarkers of progression. (C) Heatmaps depicting subgroup-specific lipid profiles, with representative alterations observed in stage, tumor size, pathology, LN metastasis, and distant metastasis. Early stage was defined as AJCC 8th edition stage I–II and late stage as stage III–IV. Regional LN involvement denotes nodal status (N0 *vs* N+). Distant metastasis denotes metastatic status at diagnosis (M0 *vs* M1). (D) Representative serum lipids show systematic associations with tumor burden and metastatic status.

To investigate subgroup-specific lipidomic variations, differential lipids associated with tumor stage, tumor size, histological subtype, lymph node metastasis, and distant metastasis were analyzed. UpSet plots demonstrated both unique and shared lipid alterations across subgroups. Histological subtype showed the largest number of differential lipids, followed by tumor size and lymph node metastasis, suggesting stronger influences of these clinical factors on lipid metabolic reprogramming. Venn diagram analysis revealed overlapping differential lipids across subgroups, among which SM 32:2;2O was shared by both lymph node and distant metastasis subgroups, suggesting its potential as a core biomarker associated with metastatic progression (Figs. 7A–7B). In the volume-stratified analysis, PS(O-36:2) was higher in large tumors (≥5 cm) than in small tumors (≤5 cm), indicating a size-dependent increase (FDR < 0.05) (Fig. 7D). These subgroup findings suggest that serum lipid alterations may vary across clinicopathological subsets of NSCLC.

## Discussion

In this study, we applied serum lipidomics using HPLC-HRMS to characterize lipid alterations in NSCLC patients relative to healthy controls. By integrating these data with cross-validated machine-learning analyses, we identified candidate discriminatory lipids and generated a basis for further validation and biological investigation.

At the serum level, we observed a broad downward shift in normalized intensities across several lipid classes, most notably glycerophospholipids, sphingolipids, and triacylglycerols. This pattern is consistent with disturbances in membrane-associated lipid metabolism, with potential relevance to membrane biogenesis, signaling, and energy balance (Jiang et al., 2024; Hwang et al., 2023). At the same time, it should be interpreted with caution, because residual pre-analytical and analytical influences cannot be fully excluded. Within this context, our findings remain broadly consistent with previous lipidomic studies reporting alterations in PC, PE, and SM subclasses in NSCLC (Marien et al., 2015; Yan et al., 2024). While we observed decreased SM species, we did not identify a uniform compensatory increase in circulating ceramides across the cohort, highlighting the complexity of sphingolipid remodeling in serum and the need for targeted quantitative profiling in future studies.

**Table 2:** Discrimination and calibration performance of machine learning models in differentiating lung cancer from controls.

**Model**	**AUC** ** (95% CI)**	**Accuracy**	**F1 score**	**NPV**	**PPV**	**Sensitivity**	**Specificity**	**Brier** ** score**	**Threshold**
LASSO	0.926 (0.834–1.000)	0.957	0.963	0.966	0.951	0.975	0.933	0.069	0.646
LightGBM	0.956 (0.882–1.000)	0.914	0.923	0.875	0.947	0.900	0.933	0.074	0.577
SVM	0.947 (0.878–1.000)	0.943	0.950	0.933	0.950	0.950	0.933	0.075	0.582
XGBoost	0.951 (0.895–1.000)	0.929	0.937	0.903	0.949	0.975	0.933	0.074	0.493

**Notes.**

AUC area under the receiver operating characteristic curve Accuracy proportion of correctly classified cases Sensitivity true positive rate Specificity true negative rate PPV positive predictive value NPV negative predictive value F1 score harmonic mean of sensitivity and PPV Brier score mean squared error between predicted probabilities and observed outcomes (range: 0–1, with lower values indicating better calibration)

**Table 3:** Representative lipid biomarkers identified through LightGBM-RFE modeling.

*Name*	*Formula*	*m/z*	*RT (min)*	*ppm*	*Reference Ion*	*log2FC*	*P value*
SL 38:5	C38H67NO6S	666.4787	7.028	3.9	[M+H]+	−0.90	1.30E−03
LPC(O-18:1)	C26H54NO6P	508.3768	6.161	1.18	[M+H]+	−1.23	8.27E−07
ST 27:1;O;S	C27H46O4S	465.3045	7.744	0.22	[M-H]-	−1.23	8.38E−07
Decanoylcarnitine	C17H33NO4	316.2483	1.23	0.04	[M+H]+	−0.90	9.35E−04
LPE(O-18:1)	C23H48NO6P	466.3295	6.787	0.66	[M+H]+	1.04	3.58E−05
Testosterone sulfate	C19H28O5S	367.1588	0.924	0.9	[M-H]-	−1.06	4.86E−05
Cer 41:1;O3	C41H81NO3	680.6204	16.658	0.36	[M+HCOO]-	−0.86	4.94E−04
LPC 22:6	C30H50NO7P	568.3405	2.64	1.35	[M+H]+	0.96	5.58E−05
LPE 16:0	C21H44NO7P	454.2925	3.498	0.92	[M+H]+	−0.78	2.85E−03
DG 34:2 (16:0/18:2)	C37H68O5	610.5408	13.748	0.49	[M+NH4]+	−1.00	5.66E−05
PA 34:4	C37H67O7P	655.4698	12.907	0.14	[M+H]+	−0.84	1.17E−03
PC(O-37:4)	C45H84NO7P	782.6052	15.391	−0.77	[M+H]+	−0.85	1.63E−03
PC(O-34:3)	C42H80NO7P	742.5744	14.842	−0.21	[M+H]+	−0.94	1.63E−04
TG 56:8 (16:0/18:2/22:6)	C59H98O6	920.7696	18.729	−0.62	[M+NH4]+	0.93	1.48E−04
DG 36:4 (18:2/18:2)	C39H68O5	634.5403	12.909	−0.32	[M+NH4]+	−0.96	1.47E−04
PC(O-36:3)	C44H84NO7P	770.6054	15.211	0.14	[M+H]+	−0.79	1.95E−03
LPC 20:5	C28H48NO7P	542.3248	2.329	1.34	[M+H]+	−0.75	2.63E−03
SM 42:4;O2	C47H89N2O6P	809.6510	15.337	−2.57	[M+H]+	−0.80	1.85E−03

Within a unified analytical framework, four supervised learning algorithms—LASSO, SVM, XGBoost, and LightGBM—were evaluated to reduce reliance on a single model (Wang et al., 2026; Shuang et al., 2024 Li et al., 2022). LightGBM achieved the best performance in this dataset and was paired with recursive feature elimination and SHAP value analysis to improve interpretability (Lundberg et al., 2020; Elguoshy, Zedan & Saito, 2025). Lipid features selected by LightGBM-RFE were then integrated with clinical covariates to construct a multivariable risk model, which showed good discrimination and calibration under internal stratified cross-validation. Decision-curve analysis suggested potential net benefit across a range of threshold probabilities, and a nomogram was constructed to facilitate individualized risk estimation (Zhang et al., 2025; Vickers et al., 2008; Balachandran et al., 2015; Steyerberg & Vergouwe, 2014). These findings are encouraging, but they remain preliminary and require external validation before any clinical application.

The three-lipid panel was biologically plausible. LPC(O-18:1) is related to phospholipid remodeling and lysophospholipid signaling, both of which have been implicated in tumor-associated membrane dynamics (Zhang et al., 2021; Magkrioti et al., 2018; Papin et al., 2024). Decanoylcarnitine reflects fatty-acid oxidation and mitochondrial metabolic balance, supporting a link between the observed serum changes and altered energy metabolism in cancer (Longo, Frigeni & Pasquali, 2016; Ni et al., 2019). The SL (38:5) feature was also decreased in serum; however, because confirmatory MS/MS was not available, this annotation should be considered putative and interpreted conservatively (Jirásko et al., 2022; Yao et al., 2019). Taken together, these lipids may capture complementary aspects of membrane remodeling and metabolic reprogramming in NSCLC.

We also observed subgroup-associated lipid differences, suggesting that serum lipid alterations may vary across clinicopathological subsets of NSCLC. For example, PS(O-16:0/20:2) increased with tumor size, whereas SM(32:2;O2) was lower in both lymphatic and distant metastatic subgroups than in the corresponding non-metastatic groups. These findings may reflect differences in lipid remodeling across disease burden and metastatic status, although the underlying mechanisms remain to be clarified (Cui et al., 2021; Benjamin et al., 2013; Dayoub & Brekken, 2020; Beckham et al., 2012; Hait & Maiti, 2017; Takanashi et al., 2020).

Several limitations merit consideration. First, this was a single-center, exploratory case-control study with a modest sample size and no independent external validation, which limits statistical power and increases the risk of overfitting when modeling high-dimensional lipidomic data (Wynants et al., 2020; Collins et al., 2015). Internal cross-validation and bootstrap-based procedures reduce optimism but cannot substitute for prospective validation in independent cohorts. Second, the NSCLC cohort included mixed stages and was enriched for advanced disease, which may introduce spectrum bias and inflate apparent discrimination relative to screening-relevant settings. Third, specificity remains a central challenge in small-sample biomarker discovery. Some lipid features, including LPC(O-18:1), have been associated with non-NSCLC conditions (Li et al., 2024; Mishra et al., 2024), and residual confounding related to general health status or systemic inflammation cannot be fully excluded. Finally, pre-analytical and analytical variability—including differences in fasting compliance, sample handling, storage, and platform-specific coverage—may have contributed to the observed class-wide downward trend and may also affect detectability of particular lipid subclasses (Kirwan et al., 2019; Burla et al., 2018). Future studies should therefore prioritize standardized workflows, targeted quantitative validation of prioritized lipids, and mechanistic investigation in larger, independently collected cohorts (Contrepois et al., 2018).

## Conclusions

This exploratory study integrated HPLC-HRMS serum lipidomics with interpretable machine learning to develop a diagnostic framework for discriminating NSCLC cases from controls. Under internal cross-validation, the combined lipid-clinical model showed encouraging discrimination and calibration within this cohort, and SHAP-based analyses improved transparency of the prioritized lipid features. We also observed broad serum lipidomic differences between NSCLC patients and healthy controls, with a predominance of lower-abundance lipids in the NSCLC group. However, given the exploratory design, modest sample size, and the possibility of residual technical or pre-analytical influences, these findings should be interpreted with caution and require targeted validation in larger, independently collected cohorts.

##  Supplemental Information

10.7717/peerj.21504/supp-1 Supplemental Information 1 Supplemental figures

10.7717/peerj.21504/supp-2 Supplemental Information 2 The analysis code used in this study

10.7717/peerj.21504/supp-3 Supplemental Information 3 Lipid dataset

10.7717/peerj.21504/supp-4 Supplemental Information 4 OPLS-DA script

10.7717/peerj.21504/supp-5 Supplemental Information 5 Supplementary feature matrix

## Additional Information and Declarations

### Data Availability

The following information was supplied regarding data availability: The data and code are available in the Supplemental Files. The raw LC–MS instrumental data are available at Mendeley Data: Tang, Chengxi (2026), “HPLC-HRMS and Interpretable Machine Learning Decipher Serum Lipidomic Signatures in NSCLC”, Mendeley Data, V1, doi: 10.17632/rgypd4sjy7.1
The processed dataset used for statistical analyses is available at Mendeley Data: Tang, Chengxi (2026), “HPLC-HRMS and Interpretable Machine Learning Decipher Serum Lipidomic Signatures in NSCLC”, Mendeley Data, V2, doi: 10.17632/85ywdvx66y.2.
